# Molecular diagnostics for congenital hearing loss including 15 deafness genes using a next generation sequencing platform

**DOI:** 10.1186/1755-8794-5-17

**Published:** 2012-05-18

**Authors:** Sarah De Keulenaer, Jan Hellemans, Steve Lefever, Jean-Pierre Renard, Joachim De Schrijver, Hendrik Van de Voorde, Mohammad Amin Tabatabaiefar, Filip Van Nieuwerburgh, Daisy Flamez, Filip Pattyn, Bieke Scharlaken, Dieter Deforce, Sofie Bekaert, Wim Van Criekinge, Jo Vandesompele, Guy Van Camp, Paul Coucke

**Affiliations:** 1 NXTGNT, Ghent University, Ghent, Belgium; 2 Center for Medical Genetics, Ghent University, Ghent, Belgium; 3 Biobix, Laboratory for Bioinformatics and Computational Genomics, Ghent University, Ghent, Belgium; 4 Laboratory for Pharmaceutical Biotechnology, Ghent University, Ghent, Belgium; 5 Department of Medical Genetics, University of Antwerp, Antwerp, Belgium; 6 Department of Medical Genetics, School of Medicine, Ahvaz Jundishapur University of Medical Sciences, Ahvaz, Iran

**Keywords:** Deafness, Next generation sequencing, PCR based enrichment, Genetic diagnostics

## Abstract

**Background:**

Hereditary hearing loss (HL) can originate from mutations in one of many genes involved in the complex process of hearing. Identification of the genetic defects in patients is currently labor intensive and expensive. While screening with Sanger sequencing for *GJB2* mutations is common, this is not the case for the other known deafness genes (> 60). Next generation sequencing technology (NGS) has the potential to be much more cost efficient. Published methods mainly use hybridization based target enrichment procedures that are time saving and efficient, but lead to loss in sensitivity. In this study we used a semi-automated PCR amplification and NGS in order to combine high sensitivity, speed and cost efficiency.

**Results:**

In this proof of concept study, we screened 15 autosomal recessive deafness genes in 5 patients with congenital genetic deafness. 646 specific primer pairs for all exons and most of the UTR of the 15 selected genes were designed using primerXL. Using patient specific identifiers, all amplicons were pooled and analyzed using the Roche 454 NGS technology. Three of these patients are members of families in which a region of interest has previously been characterized by linkage studies. In these, we were able to identify two new mutations in *CDH23* and *OTOF*. For another patient, the etiology of deafness was unclear, and no causal mutation was found. In a fifth patient, included as a positive control, we could confirm a known mutation in *TMC1.*

**Conclusions:**

We have developed an assay that holds great promise as a tool for screening patients with familial autosomal recessive nonsyndromal hearing loss (ARNSHL). For the first time, an efficient, reliable and cost effective genetic test, based on PCR enrichment, for newborns with undiagnosed deafness is available.

## Background

Hearing loss (HL) is the most common birth defect in industrialized countries and the most prevalent sensorineural disorder. One out of every 500 newborns has bilateral permanent sensorineural HL of more than 40 dB HL [1]. It is estimated that in developed countries, genetic causes are responsible in at least two-thirds of prelingual cases. In most of the cases there are no clinical abnormalities other than the hearing loss (i.e. nonsyndromic hearing loss, NSHL). Inherited NSHL is monogenic, with over 100 mapped loci and 46 causally implicated genes [2].

*GJB2* mutations are the most frequent cause of autosomal recessive non-syndromic hearing loss (ARNSHL) and account for about 20 % of the cases [3]. Therefore, newborns that are diagnosed with severe-to-profound HL in the absence of other abnormal findings on physical examination are analyzed for mutations in the *GJB2* gene. In some cases, when imaging of the inner ear shows abnormalities such as an enlarged vestibular aquaduct, the *SLC26A4* gene is analyzed. Besides these genes there is hardly any other gene that is routinely analyzed in DNA diagnostics. For this reason, a positive result is only obtained in less than 20 % of deaf children for which DNA diagnostics is requested [4]. While substantial progress has been made in recent years in identifying the responsible deafness genes, the key challenge lies in determining which gene is responsible in a patient. Sequencing of all genes by traditional DNA sequencing technology is labor intensive and not cost effective [5].

Recently the next generation DNA sequencing technology has come of age [6]. Companies such as Roche (454 Genome Sequencer FLX), Applied Biosystems (Solid System) and Illumina (Genome Analyzer) have brought high throughput DNA sequencers to the market that can sequence several hundred million to a few billions of basepairs in a few days. Until now, this technology has mainly been used for research purposes. Several genes for hearing loss have been identified using this technology [7][8]. Most likely, the identification of other as yet unidentified deafness genes will follow. On the other hand, applications in DNA diagnostics in general are still rare. A first genetic test encompassing the NSHL genes using massively parallel sequencing technology has been described [5][9]. In these studies, an array-based enrichment approach was used on a limited number of patients. Although this new technology holds the promise to significantly reduce the cost and workload per sample, the enrichment used leads to a significant loss in sensitivity, which is deemed unacceptable according to current standards using PCR amplification. Array based enrichment procedures inherently suffer from incomplete selection for two reasons. Firstly, due to the presence of repetitive sequences, not all fragments can be included in the selection set. Secondly, hybridization based enrichment suffers from selection bias and uneven capture efficiency. Much deeper sequencing will be needed to ensure complete or sufficient coverage of the selected fragments, in comparison to unbiased selection techniques such as PCR. In combination, these factors lead to a reduced sensitivity, while sensitivity requirements for DNA diagnostics are generally required to be very high. Here, we report the evaluation of a PCR based enrichment strategy followed by a 454 NGS approach for 5 patients using NGS implicated in ARNSHL.

## Results

Five patients with familial congenital deafness were screened for 15 deafness genes on the 454 Genome Sequencer in order to evaluate if next generation sequencing enables the detection of mutations. Three of these patients are members of families in which a region of interest has previously been characterized by linkage studies. Therefore, mutations in respectively *CDH23* (for patients 1 and 2) and *OTOF* (for patient 4) were expected. In patient 5, a known mutation (c.236 + 1 G > A) in *TMC1* had to be confirmed. For patient 3, no mutation was known.

We chose the Roche 454 next generation sequencing platform because of the longer read lengths (up to 400 bp) of the pyrosequencing approach. We decided to screen the 15 most important autosomal recessive deafness genes, selected on their reported mutation frequency [4] (Table 1). By limiting the number of genes to 15, we estimated to analyze 10 to 15 patients within a single sequencing run [10].

**Table 1:** Analyzed genes

**Gene**	**Number of exons**	Number of mutations worldwide**	Number of homopolymer repeats* in CDS	**Function in hearing process**
*GJB2*	2	> 220	0	ion homeostasis
*SLC26A4*	21	43	7	ion homeostasis
*MYO15A*	66	28	8	hair bundle, motor protein
*OTOF*	48	26	4	exocytose at auditory ribbon synapse
*CDH23*	69	21	4	hair bundle, adhesion protein
*TMC1*	24	20	4	unknown function
*TMPRSS3*	13	16	3	unknown function
*TECTA*	23	10	2	extracellular matrix protein
*TRIOBP*	24	9	7	hair bundle, cytoskeletal formation
*TMIE*	4	8	0	unknown function
*PJVK*	7	7	3	signaling of hair cells and neurons
*ESPN*	13	6	1	hair bundle, cytoskeletal formation
*PCDH15*	33	5	8	hair bundle, adhesion protein
*ESRRB*	12	5	1	transcription factor
*MYO7A*	49	5	5	hair bundle, motor proteins

(*) Homopolymer repeats ≥ 6 located in the exons +/− 10 bp.

(**) Reference: [4].

**Figure 1:**
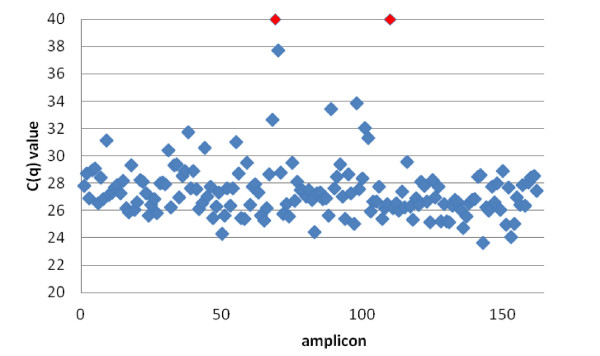
**Cq values for *OTOF* and *CDH23* .** The Cq value is displayed in function of the corresponding amplicon for the *OTOF* and *CDH23* gene. The plot shows a drop-out of two amplicons (red).

**Figure 2:**
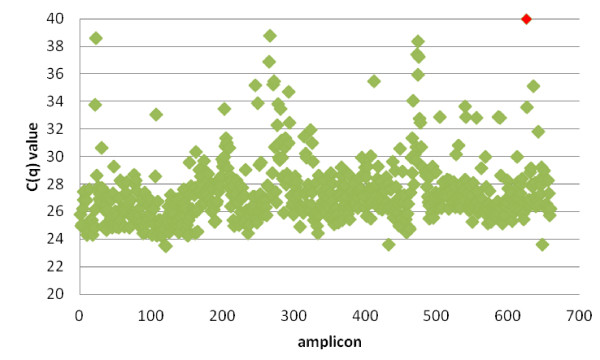
**Cq values for the 15 genes.** The Cq values for the amplicons covering all of the 15 genes are displayed. One amplicon of the *TRIOBP* gene failed during this PCR (red).

The enrichment protocol prior to the sequencing was based on an individual PCR amplification of all amplicons covering the coding region of the 15 genes. A prerequisite to use this PCR approach is that the reaction conditions for all the amplicons are uniform, enabling a semi-automated workflow by using 384-well plates and liquid handling robots. In a pilot experiment, we designed 162 forward and reversed primers for the coding regions of *OTOF* and *CDH23*, using our in-house developed primer design software package primerXL, in order to evaluate the uniformity of the reaction conditions. Amplification was performed on normal genomic DNA (Roche diagnostics) and all amplicons, except 3, could be amplified. An average Cq of 27 was obtained (Figure 1). The 3 failed amplicons were redesigned. Subsequently, primers were designed for the coding regions of the remaining 13 genes. In the end, a set of 646 primer pairs covering all the coding sequences and most of the UTRs of the 15 deafness genes in our setup was developed (Figure 2).

In the next step, the patients’ DNA samples were amplified. Initially, the PCRs for patients 1, 2 and 3 were performed. These patients were analyzed with the standard chemistry on a full PicoTiterPlate using 2 large regions (Table 2). The total number of reads after filtering and mapping was 325.272, with an average read length of 250 bp. The resulting mean coverage was 221x (patient 1), 168x (patient 2) and 194x (patient 3). Approximately 95 % of the screened amplicons had a coverage above 38, which makes them available for variant analysis (Table 2) [10]. As such high average coverage is not necessary for reliable variant analysis; the next run was modified accordingly.

**Table 2:** Sequencing results

	**Patient 1**	**Patient 2**	**Patient 3**	**Patient 4**	**Patient 5**
**Number of NGS runs**	1	1	1	2	2
**454 experiment**	Standard 1/3 run	Standard 1/3 run	Standard 1/3 run	Standard 1/5 Titanium 1/12	Standard 1/5 Titanium 1/12
**Mapped reads**	127980	96538	111492	24940* (Std) 24192* (Tit)	16767* (Std) 40169* (Tit)
**Total average coverage**	221	168	194	73*	88*
**% sequenced amplicons with coverage > 38**	94.4	93.2	96.3	80.3	85.3
**% sequenced amplicons with coverage > 30**	95.3	94.6	97.2	86.8	91.5
**% sequenced amplicons with coverage > 5**	97.2	97.2	99.0	94.6	96.3
**Mutations**	New mutation found in *CDH23*	New mutation found in *CDH23*	No mutation could be clearly identified	New mutation found in *OTOF*	Known mutation in *TMC1* confirmed

(*) The values for the average coverage for patient 4 and 5 are the total over the 2 runs (standard and titanium).

For patients 4 and 5, 1/5 of the capacity of a Standard run with 2-lane gasket was used for each patient. The average coverage was lower than expected and therefore an additional Titanium run (1/12) was performed to have extra reads (Table 2), resulting in an average total coverage of 73 and 88 for respectively patient 4 and patient 5 (Table 2).

A novel missense mutation in *CDH23* could be identified for patients 1 and 2, a result that is in agreement with the convincing linkage data previously found (data not shown). Both are homozygous for c.5527 G > T resulting in an amino acid substitution from Asp to Tyr at position p.1843 in exon 43. The mutation was found in 241 reads out of 241 and in 101 reads out of 101 for patient 1 and 2 respectively. Subsequently, the mutation was confirmed with Sanger sequencing. The mutation is classified as probably damaging by Polyphen, is not tolerated by SIFT (Table 3) and was not reported before, nor as disease causing mutation nor as a SNP. The mutation was analyzed in the whole family and showed full co-segregation with the phenotype.

**Table 3:** New variants observed in patients 1, 2 and 4

**Variants**	**Prediction (PolyPhen-2)**	**Prediction (SIFT)**	**Prediction (AGVGD)**	**Grantham score ([0–215])**
**Patient 1–2 *CDH23* NM_022124.4:c.5527 G > T Chr10(NCBI 36):g.73214678 G > T p.Asp1843Tyr**	Probably damaging	Not tolerated	Most likely interfere with function (Class C65)	160
**Patient 4**				
** *OTOF* NM_194248.2:c.3263 T > C Chr2(NCBI 36):g.26550910 T > C p.Leu1088Pro**	Possibly damaging	Not tolerated	Less likely interfere with function (Class C0)	98

*Source:* Alamut version 1.53 (Interactive Biosoftware).

In patient 3, we couldn’t identify a mutation that is undeniably disease causing. After filtering all the variants, obtained with data analysis and filter settings as described in ‘Methods’, we obtained a list of 171 variants. Ninety-seven of these are located in the introns (further than 10 basepairs away from the exonic regions). Another set of 22 variants is located in the UTRs and are all reported as polymorphisms. Of the 171 variants, 48 of them are previously reported as polymorphism or were identified in several patients in the lab. Eventually, 7 unique variants remained. Five of them are synonymous SNPs with no effect on splicing based on splice prediction programs. One deletion (c.3636_3637del) located in the *OTOF* gene, results in a frame shift (p.Phe1212fs). This mutation could not be confirmed with Sanger sequencing (see Table 4). The second mutation results in an amino acid substitution in the *CDH23* gene and was found in 50 out of 96 reads. This variant is not known as a polymorphism. However, it’s unclear that this missense mutation is causal as the prediction tools are not conclusive (see Table 4). No additional mutation was found in the *CDH23* gene. Further evaluation of the known polymorphisms revealed a low population frequency for 4 of them. Although all were reported as non-pathogenic, we took a closer look at these. One variant occurs in the *TMPRSS3* gene with a relative frequency in the reads of 1, previously reported as rs35227181 and considered to be non-pathogenic. Also the prediction tools didn’t classify the variant as disease causing and therefore we considered this base change as a rare variant. In *OTOF *a compound heterozygous variant was found: c.2317 C > T (p.Arg773Cys) and c.4936 C > T (p.Pro1646Ser). Both variants were detected with a relative frequency of 0.5 and are predicted to be possibly damaging (Table 4). The two variants were previously described in the literature, where they were considered as non-pathogenic [11][12]. Another variant was found in *PCDH15* in 380 out of 381 reads. The variant is known as rs4935502 and non-pathogenic according to dbSNP. Nonetheless, it is regarded as probably damaging by PolyPhen-2 and has a Grantham score of 126 (see Table 4). All the homopolymer repeats of these deafness genes (Table 1) were reanalyzed with Sanger sequencing since the 454 technology does not allow reliable sequencing of repeats greater than 6. No mutation could be detected in this analysis. It remains possible that the mutation is located in another deafness gene, not yet incorporated in our set, or that patient 3 is a non-genetic deafness case.

**Table 4:** Variants observed in patient 3

**Gene**	**Variant**	**Relative frequency**	**PolyPhen-2**	**SIFT**	**AGVGD**	**Grantham score**	**Splicing**	**Sanger sequencing**
** *CDH23* **	**NM_022124.5:c.8167 G > C Chr10(GRCh37):g.73566027 G > C p.Val2723Leu**	0.52	Possibly damaging	Tolerated	Class C0	32	Not affected	/
** *OTOF* **	**NM_194248.2:c.3636_3637del Chr2(NCBI36):g.26549600_26549601del p.Phe1212fs **	0.37	/	/	/	/	Frame shift (The new reading frame ends in a STOP codon 78 positions downstream.)	Not confirmed
	**NM_194248.2:c.2317 C > TChr2(NCBI36):g.26553877 C > T p.Arg773Cys rs80356569**	0.52	Probably damaging	Not tolerated	Class C25	180	Not affected	Confirmed
	**NM_194248.2:c.4936 C > T Chr2(NCBI36):g.26541265 C > T p.Pro1646Ser rs17005371**	0.48	Benign	Not tolerated	Class C65	74	Not affected	Confirmed
** *PCDH15* **	**NM_001142763.1:c.1319A > C Chr10(NCBI36):g.55625450A > C p.Asp440Ala rs4935502**	1	Probably damaging	Tolerated	Class C0	126	Not affected	Confirmed

The variant analysis of patient 4 revealed a new homozygous missense mutation in the *OTOF* gene (exon 26: c.3263 T > C (p.Leu1088Pro)). This mutation is in agreement with linkage analysis, previously performed, suggesting a disease causing mutation in the *OTOF* gene. Prediction programs revealed the mutation as possibly damaging (PolyPhen) and not tolerated (SIFT) and therefore can most likely be considered as disease causing. The mutation was investigated in all available family members and showed full co-segregation with the deafness. The prediction results of the novel mutations, with the different software approaches, are listed in Table 3.

In patient 5, included as a control, we could confirm the heterozygous mutation in the *TMC1* gene previously found with Sanger sequencing, with a relative variant frequency of 0.5. The mutation NM_138691.2:c.236 + 1 G > A was found in 13 reads out of 26. This substitution is located in the donor splice site of intron 7.

## Discussion

We have developed an assay to improve the molecular diagnosis of autosomal recessive nonsyndromic hearing loss (ARNSHL) by simultaneous sequencing of the exons, UTRs and alternative transcripts of 15 deafness genes. The selected list of genes includes the most frequently mutated recessive genes in patients with hearing loss (Table 1). In contrast to hybridization based capture approaches [5], we performed a PCR based enrichment strategy for all target regions of the 15 genes followed by sequencing with a 454 Genome Sequencer FLX. The optimization of the PCR amplification conditions made an efficient semi-automated high throughput processing of the many PCR reactions feasible and straightforward. The PCR reactions of all difficult amplicons can easily be repeated and sequenced with the conventional Sanger method. We are able to complete the screening in a relatively short period of time. The adapter ligation method (Shotgun protocol) was preferred over the fusion primer approach for amplicon sequencing, in order to reduce the sequencing cost and to obtain a more efficient workflow. When screening a limited number of genes, it is more advantageous to use gene specific primers with the forward and reverse adapters already incorporated in the oligo’s. For the 15 genes (646 amplicons) in our setting, the fusion primer approach is cost prohibitive, since the adapter sequence needs to be incorporated in every single primer set. Therefore, the adapters were ligated in one reaction to the pool of amplicons.

Two new mutations were discovered and a known heterozygous mutation could be confirmed. The depth of sequencing coverage was high (greater than 38) for over 90 % of the amplicons, indicating that the majority of amplicons are covered sufficiently. Sanger sequencing remains indispensable to confirm results, to analyze homopolymer regions and to sequence drop-out amplicons. Since this was a pilot study, we restricted the screening to 5 patients. However, by this approach we are able to screen 15 patients within a single Titanium run, making this workflow cost-effective. While further validation with a larger panel of positive controls is needed, our approach holds great promise. Although it was an advantage that the region of interest had already been localized by linkage analysis for patients 1–2 and 4; we believe that we would have found the mutation without the knowledge of the linked region. The main reason for this is that there were no other relevant mutations found in the remaining 14 analyzed genes that could be causal. The new variants that we found in *CDH23* and *OTOF* were homozygous with a relative frequency of 1. These mutations in *CDH23* and *OTOF* were only found in respectively patients 1–2 and 4 and not in the other analyzed deafness samples (e.g. we often did find the same SNP’s in different patients). At the same time, there was no doubt about the causality of both mutations as predicted by the different software tools.

Genetic counseling prior to analysis of genes potentially revealing Usher syndrome (*CDH23, PCDH15 *and* MYO7A*) is important and this should be explained to parents who agree to have this type of diagnostics performed for their child.

With patient 3, in whom we were not able to determine the disease causing mutation, we illustrate that the major difficulty with this kind of analysis is the interpretation of the variants. In some cases, the disease causing nature of the variant will not be convincing and additional investigations as segregation in the family or functional analysis will be essential.

## Conclusions

Our data demonstrate that the use of NGS technology holds promises as a tool for screening congenital deafness genes. The availability of a more profound diagnostic test compared to the actual “gene by gene” analysis approach, will provide better opportunities to identify the disease causing mutation in patients permitting prompt management and accurate genetic counseling. Once a substantial number of patients originating from a specific population are analyzed, we will be able to determine the relative contribution of this set of 15 deafness genes to recessive hearing loss in this specific population, data which is currently unavailable. From a technical point of view, the screening can be performed even on a sporadic case. However less positive results will be obtained since there are a significant amount of deafness cases caused by non-genetic factors. A continued refinement of the NGS technology will further improve the sequencing accuracy and reduce the cost. At the same time bio-informatics tools will improve. This will be critical for the interpretation of NGS-data and essential for the diagnostic laboratories using NGS technology.

## Methods

### Patient material

Genomic DNA was obtained from five patients with congenital HL. Patient 1 and 2 are the probands of a consanguineous recessive Iranian family with at least 9 affected members for which linkage to the *CDH23* locus has been found previously. Patient 3, a member of a Turkish family, has congenital deafness. This patient has parents with normal hearing and *GJB2* mutations have been excluded. Patient 4 is the proband of a consanguineous Iranian family with recessive deafness, for which linkage to the *OTOF* locus has been proven. The family had 7 affected members. Both Iranian families were seen by a geneticist and filled in a general clinical questionnaire. However, no detailed audiometric or ophthalmological examinations were performed, because the families were collected in remote locations. Both families suffered from profound early childhood hearing loss and no obvious signs of syndromal hearing loss were noted. In patient 5, a known mutation in the *TMC1* gene was identified previously, and this patient served as a positive control. As patient 3 was part of routine deafness screening, specific ethical approval was not required. Both Iranian families are part of a research project at the University of Antwerp, for which ethical approval was obtained.

### Primerdesign and amplicon PCR

Fifteen autosomal recessive deafness genes were selected, based on their reported mutation frequency (Table 1) [4]. The genes with the highest frequency of mutations reported in the literature were chosen. Primer design using the in house developed primerXL pipeline (Lefever et al., in preparation), resulted in 646 oligonucleotide pairs covering all the coding sequences (CDS) and most of the UTRs of the 15 genes responsible for ARNSHL. After the first round of primer design, with the most stringent conditions (no SNPs in primer annealing region, amplicon length between 250–350 bp, GC content between 30 and 80 %), 97.1 % of all the regions of the 15 genes could be amplified successfully. The missing regions were caused by systematic drop-out of some amplicons during PCR. The primer design was optimized in different steps by accepting less stringent conditions; first by tolerating an increase in the number of generated primers (for example to allow the presence of a single SNP in the 5’ region of the primer), then by lowering the permitted amplicon length and finally by slightly varying the melting temperature (Tm) of the primers. All PCR reactions are performed using the same reaction conditions, enabling an automated workflow. The average length across the 646 amplicons is 319 bp, resulting in an aggregate target size of approximately 200 Kb. The primers used in this step are modified at their 5' end with a universal M13 linker sequence. The primer sequences were synthesized by Integrated DNA technologies and delivered in 384-well plates as a 100 μM stock solution (forward and reverse primer separately).

All 646 amplification reactions of the 15 genes were carried out as singleplex PCR in two 384-well plates per patient. A master mix for each sample was prepared and consisted of 1x Kapa Taq buffer (Sopachem), 1 mM MgCl_2_ (Roche Diagnostics), 0.12 mM dNTP’s (Invitrogen), 0.02 U/μl Kapa Taq polymerase (Sopachem), 0.32x LC Green Plus (Bioké) and 25 ng gDNA per reaction. Then, 1.25 μl of the forward/reverse primer mix (1 μM stock solution) was added to the 8.75 μl master mix using a Freedom EVO Tecan liquid handling robotic workstation, resulting in a final volume of 10 μl per reaction. The PCRs were first tested on Human Genomic DNA (Roche diagnostics). The amplification reactions were carried out on a C1000 real-time thermal cycler (Bio-Rad) with following cycling conditions: 95°C-5’, 94°C-30”, 58°C-30”, 72°C-50”; 40 cycles (+ plate read after each one), followed by a melt curve (65°C > 95°C for 5” increment 0.5°C). The Cq value was determined and the size of the amplified products was verified on a MultiNA (Shimadzu Biotech). To obtain an equal representation of every amplicon in the pool, PCR products were pooled in an equimolar manner for each patient, based on the end-point fluorescent values. Subsequently, 100 μl of these pools were purified with the High Pure PCR Cleanup Micro Kit (Roche Diagnostics). The quality of the PCR pools was verified on an Agilent 2100 Bioanalyzer with the DNA 1000 K chip (Agilent technologies) and the concentration was measured with the Quant-it Picogreen DNA assay (Invitrogen).

### Next generation sequencing

**Figure 3:**
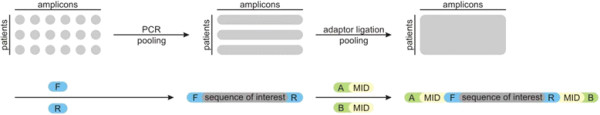
**Workflow for the PCR enrichment approach.** Amplicons are equimolar pooled per patient, continuously the A and B (454) adapters with MID are ligated to each pool of amplicons. All the patients, each tagged with a unique MID, can be pooled before proceeding to emulsion PCR.

The Roche A and B adapters with a MID (Multiplex IDentifier), were ligated to each purified pool of amplicons according to the Shotgun protocol for Low Molecular Weight (LMW) samples (Manual: ‘GS FLX Shotgun DNA Library Preparation Method Manual’ (December 2007), skipping the fragmentation step. A different MID sequence was used for every patient (Figure 3). Quality control of the library preparation was carried out on a RNA 6000 pico labchip (Agilent technologies) and concentration of the library was measured with the Quant-it Ribogreen RNA assay (Invitrogen). Emulsion PCR and pyrosequencing on the 454 Genome Sequencer FLX (GS FLX) were carried out as described (Titanium emPCR method manual, Titanium sequencing method manual Roche).

### Sanger sequencing homopolymers

We identified the homopolymer regions, defined as 6 or more repeats of the same base, located in the exonic regions of the 15 deafness genes (Table 1). These regions were analyzed with the conventional Sanger sequencing method for patient 3, since we couldn’t confirm a causal mutation in this patient.

### Data analysis

Mapping of the sequenced reads was performed by BLAT [13], software that has been integrated in V.I.P. (Variant Identification Pipeline) [14]. Variants were identified using the variant identification module included into V.I.P. with filter settings: homopolymers < 7, Quality score ≥ 30, relative variant frequency ≥ 0.35 and total coverage ≥ 20. The novel identified variants were analyzed by Alamut version 1.53 (Interactive Biosoftware).

## Abbreviations

ARNSHL, Autosomal recessive non-syndromic hearing loss; Bp, Basepairs; CDS, Coding sequences; GS FLX, Genome Sequencer FLX; HL, Hearing loss; LMW, Low molecular weight; MID, Multiplex Identifier; NGS, Next generation sequencing; PCR, Polymerase chain reaction; PTP, Picotiterplate; SNP, Single nucleotide polymorphism; UTR, Untranslated region; VIP, Variant identification pipeline.

## Competing interests

The authors declare that they have no competing interests.

## Authors’ contributions

SL designed the primers. FP contributed to the primer design. JH and SDK validated the primers. JR, HVDV and SDK performed the practical work. JDS analyzed the data. MAT and GVC performed the linkage studies. PC and SDK interpreted the data and wrote the paper. GVC contributed to the manuscript writing. JPR contributed in a major way to the optimization of the workflow. JH, FVN, DF, BS, DD, SB, WVC, JVDS, GVC and PC performed the initial study design. All authors read and approved the final manuscript.

## Pre-publication history

The pre-publication history for this paper can be accessed here:

http://www.biomedcentral.com/1755-8794/5/17/prepub
